# Use Intention and User Expectations of Human-Supported and Self-Help eHealth Interventions: Internet-Based Randomized Controlled Trial

**DOI:** 10.2196/38803

**Published:** 2024-02-15

**Authors:** Talia R Cohen Rodrigues, Thomas Reijnders, Linda D Breeman, Veronica R Janssen, Roderik A Kraaijenhagen, Douwe E Atsma, Andrea WM Evers

**Affiliations:** 1 Health, Medical, and Neuropsychology Unit Leiden University Leiden Netherlands; 2 Department of Cardiology Leiden University Medical Center Leiden Netherlands; 3 NDDO Institute for Prevention and Early Diagnostics (NIPED) Amsterdam Netherlands; 4 Vital10 Amsterdam Netherlands; 5 Department of Psychiatry Leiden University Medical Center Leiden Netherlands; 6 Medical Delta Leiden University, Technical University of Delft, Erasmus University Rotterdam Leiden, Delft, Rotterdam Netherlands

**Keywords:** eHealth, human support, Unified Theory of Acceptance and Use of Technology, use intention, UTAUT, working alliance

## Abstract

**Background:**

Self-help eHealth interventions provide automated support to change health behaviors without any further human assistance. The main advantage of self-help eHealth interventions is that they have the potential to lower the workload of health care professionals. However, one disadvantage is that they generally have a lower uptake. Possibly, the absence of a relationship with a health care professional (referred to as the working alliance) could lead to negative expectations that hinder the uptake of self-help interventions. The Unified Theory of Acceptance and Use of Technology (UTAUT) identifies which expectations predict use intention. As there has been no previous research exploring how expectations affect the adoption of both self-help and human-supported eHealth interventions, this study is the first to investigate the impact of expectations on the uptake of both kinds of eHealth interventions.

**Objective:**

This study investigated the intention to use a self-help eHealth intervention compared to a human-supported eHealth intervention and the expectations that moderate this relationship.

**Methods:**

A total of 146 participants were randomly assigned to 1 of 2 conditions (human-supported or self-help eHealth interventions). Participants evaluated screenshots of a human-supported or self-help app–based stress intervention. We measured intention to use the intervention-expected working alliance and the UTAUT constructs: performance expectancy, effort expectancy, and social influence.

**Results:**

Use intention did not differ significantly between the 2 conditions (t_142_=–1.133; *P*=.26). Performance expectancy (*F*_1,140_=69.269; *P*<.001), effort expectancy (*F*_1,140_=3.961; *P*=.049), social influence (*F*_1,140_=90.025; *P*<.001), and expected working alliance (*F*_1,140_=26.435; *P*<.001) were positively related to use intention regardless of condition. The interaction analysis showed that performance expectancy (*F*_1,140_=4.363; *P*=.04) and effort expectancy (*F*_1,140_=4.102; *P*=.045) more strongly influenced use intention in the self-help condition compared to the human-supported condition.

**Conclusions:**

As we found no difference in use intention, our results suggest that we could expect an equal uptake of self-help eHealth interventions and human-supported ones. However, attention should be paid to people who have doubts about the intervention’s helpfulness or ease of use. For those people, providing additional human support would be beneficial to ensure uptake. Screening user expectations could help health care professionals optimize self-help eHealth intervention uptake in practice.

**Trial Registration:**

OSF Registries osf.io/n47cz; https://osf.io/n47cz

## Introduction

eHealth provides the opportunity to provide remote or automated health care support through digital tools [1]. eHealth is becoming increasingly relevant, for example, because of the physical restrictions during the recent COVID-19 outbreak [2]. During this pandemic, the demand for health care support increased too. Especially vulnerable groups experienced increased mental health difficulties [3,4], which require professional support. However, health care professionals already have a high workload and pressure [5] and, in some cases, even experience an additional workload from using eHealth [6]. Self-help eHealth interventions might provide a potential solution to these problems. Self-help eHealth interventions are defined as interventions in which automated support instead of human assistance is provided [1]. As this means that no human professionals are involved, self-help eHealth interventions are easier and cheaper to widely implement [1].

Despite these advantages, self-help interventions generally deal with low levels of adherence [7-10] and low uptake [11,12]. People generally show a higher intention to start with lifestyle changes using an intervention with additional human assistance compared to a self-help intervention [13]. While there has been extensive research on the factors contributing to nonadherence, there is a notable gap in our understanding when it comes to expectations that influence whether individuals will choose to use an intervention before starting. This information is important, as a growing number of eHealth tools are being developed and proven to be effective but hardly used [14,15]. Therefore, the aim of this study is to investigate whether there is a difference in use intentions between self-help and human-supported eHealth interventions and if user expectations influence the intention to use the intervention. If we know what expectations drive people’s intention to either use self-help or human-supported eHealth interventions, we could predict and even influence their actual uptake [16].

A possible explanation for the low use intention of self-help interventions could be the lack of a relationship with a health care professional [17]. This so-called working alliance, the degree to which a health care professional and patient is involved in a useful and collaborative working relationship [18], is an important predictor of intervention adherence and effectiveness [19,20]. People are more engaged with the intervention and motivated to work on their goals when they feel supported. This effect is not exclusive to face-to-face settings; it is also evident when internet-based human assistance is involved in the use of eHealth interventions [21,22]. It is even shown to be present in self-help eHealth interventions with automated support, using, for example, a human avatar [23-25]. Thus, people can form relationships not only with other people but also with technology [26]. Therefore, we predict that people’s expectations toward a potential future working alliance when using an eHealth intervention will influence their intention to use that intervention.

Other important expectations that may influence the use intention of human-supported and self-help eHealth interventions can be found within the Unified Theory of Acceptance and Use of Technology (UTAUT) [16]. According to this model, 3 different types of expectations explain people’s intention to start with an eHealth intervention. These UTAUT expectations are (1) performance expectancy: the extent to which someone expects that the eHealth intervention will be helpful in reaching their goals; (2) effort expectancy: the extent to which someone expects that the eHealth intervention will be easy to use; and (3) social influence: the extent to which someone expects that important others believe one should use the eHealth intervention [16]. Although the UTAUT model has been used to explain people’s intention to use eHealth in general [27,28], to our knowledge, no studies have used this model to investigate differences in people’s intention to use either human-supported or self-help eHealth interventions.

In this study, we aim to investigate (1) whether there is a difference in use intention between human-supported and self-help eHealth interventions, (2) whether the expected working alliance predicts the use intention of human-supported and self-help eHealth interventions, and (3) what UTAUT constructs predict the use intention of human-supported and self-help eHealth interventions.

## Methods

### Design and Sample

In an experiment, people were presented with a sham stress management app. In this app, people would either be supported by a human coach or by an automated coach. We decided to use a student sample, as they experience high levels of stress and could therefore benefit from an eHealth stress intervention [29], especially given their increased need for support during the COVID-19 pandemic [3,4]. They were asked to evaluate the screenshots of the app and measure their use intention, the 3 UTAUT constructs (performance expectancy, effort expectancy, and social influence), and their expected working alliance. We used a randomized between-participants design with 2 experimental conditions (human-supported or self-help eHealth interventions). Healthy participants aged 18 years or older, who had a sufficient level of grasp in English, were recruited on the campus of Leiden University with internet-based and offline flyers. Power calculations [30] identified a minimum sample size of 119 to detect a medium effect (*f*^2^=0.15) with an α of .05, based on a linear multiple regression with 3 predictors.

### Procedure and Manipulation

**Figure 1:**
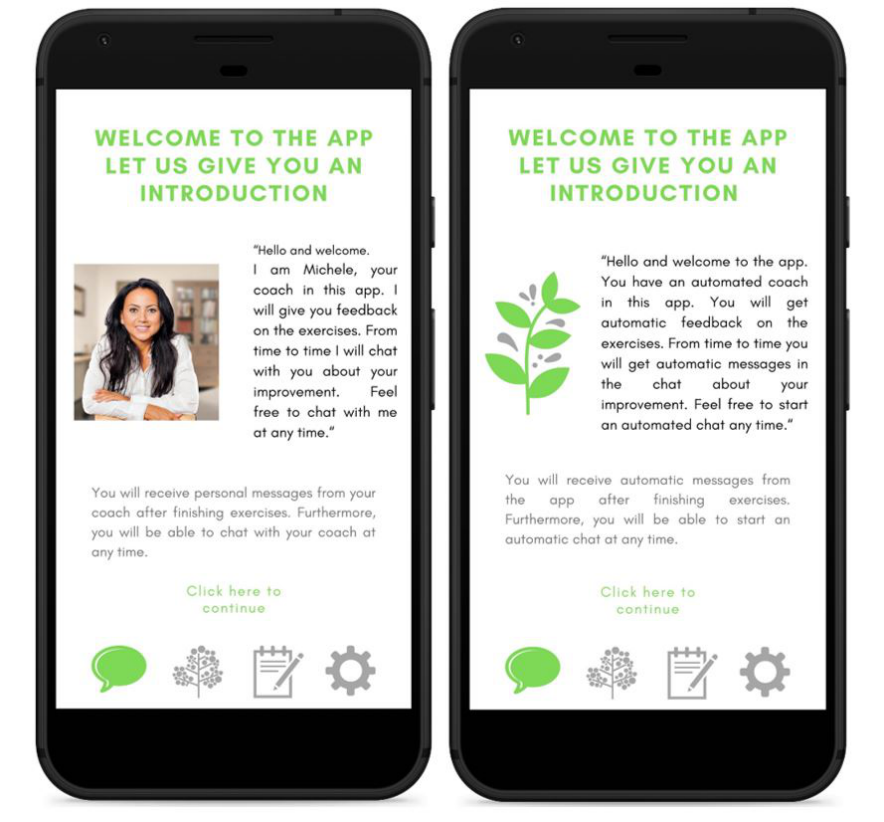
Example screenshot of the app for human-supported (left) and self-help conditions (right).

Interested participants could open the internet-based questionnaire and would be offered the internet-based consent form. After reading and agreeing to the informed consent, participants were automatically randomized into 1 of 2 experimental conditions (human-supported or self-help eHealth interventions). In both conditions, participants were instructed to evaluate a nonexistent stress management app for students called “Bye Bye Stress.” They were asked to carefully assess the screenshots of the app and give feedback to help the researchers make the app fit the needs of students. Although the design of the app and the content of the intervention were identical in both conditions, the conditions differed in the type of support that would be offered in the app. In the human-supported condition, the description of the app explained how a human coach would support the participants and provide them feedback. The screenshots of the app showed a picture of a human coach and messages with a human tone of voice (Figure 1). In the self-help condition, the description of the app explained how participants would receive automated feedback. In the screenshots, there was no picture of a human being, and the messages had a neutral tone of voice (Figure 1). All screenshots used in both conditions can be found in Multimedia Appendix 1. After this, participants were asked to complete the questionnaire.

### Measures

#### Use Intention

The behavioral intention subscale of the UTAUT questionnaire [16] was used to assess use intention. The subscale consists of 3 items (eg, “I would intend to use ‘Bye Bye Stress’ in the next 6 months.”) measured on a Likert scale ranging from 1 (strongly disagree) to 5 (strongly agree). A higher score indicates a higher intention to use the app. The scale showed a high internal consistency (Cronbach α=.953).

#### Expected Working Alliance

The expected working alliance was measured with an adjusted version of the Working Alliance Inventory–Short Revised form (WAI-SR) [31], which consists of 12 items measured on a 5-point Likert-type scale ranging from 1 (seldom) to 5 (always). Questions were adjusted to fit the context of the study by using the words “coach,” “lifestyle,” and “intervention” and being written in the future tense (eg, “The coach and I will collaborate on setting lifestyle goals.”). A higher score indicates a stronger expected working alliance. The adjusted version had a high internal consistency (Cronbach α=.917).

#### Performance Expectancy, Effort Expectancy, and Social Influence

The constructs predicting behavioral intention according to the UTAUT model—performance expectancy, effort expectancy, and social influence—were measured with the corresponding UTAUT subscales [16]. Each subscale consisted of 4 items (eg, “I find ‘Bye Bye Stress’ useful.”), measured with a Likert scale ranging from 1 (strongly disagree) to 5 (strongly agree). A higher score indicates a higher expectation of the app’s efficacy in helping the participant, a higher expectation toward the ease of use of the app, and a higher expectation that important others will approve the use of the app. The performance expectancy, effort expectancy, and social influence subscales all had sufficient internal consistency (Cronbach α of .764, .730, .792, respectively).

#### Manipulation Check

To assess whether participants carefully read the information and whether the manipulation had worked, they were asked to complete a manipulation check question (“During the intervention, I would be supported by...” followed by several options, such as “doctor” or “chatbot”).

### Analyses

To test whether there was a difference in use intention between conditions, we ran a 2-tailed independent-sample *t* test with use intention as the dependent variable and condition (human-supported vs self-help eHealth interventions) as the independent variable. To test whether the association between condition and use intention differed for different levels of the working alliance, we conducted a univariate general linear model (GLM) analysis with interactions. We added use intention as the dependent variable, condition as a fixed factor, and expected working alliance as a covariate. We analyzed both the main effects of condition and expected working alliance, as well as their interaction effect on use intention. To further investigate the interaction patterns found in the data, we conducted a simple slopes analysis. To formulate the simple slope equations for both the human-supported condition and the self-help condition, the intercept and the slope were obtained from the parameter estimates of the GLM analysis testing the association between expected working alliance and use intention.

To test whether the association between condition and use intention differed for different levels of the UTAUT constructs of performance expectancy, effort expectancy, and social influence, we conducted 3 univariate GLM analyses with interactions. We added use intention as dependent variable, condition as fixed factor, and each of the UTAUT constructs (performance expectancy, effort expectancy, or social influence) as a covariate in 3 separate analyses. We analyzed both the main effects of condition and the UTAUT construct, as well as their interaction effect on use intention. To further investigate the interaction patterns found in the data, we again conducted 3 simple slopes analyses: the intercept and the slope were obtained for both conditions from the parameter estimates of the GLM analyses testing the association between the UTAUT construct and use intention.

Statistical analyses were conducted with SPSS (version 26; IBM Corp) with a significance level set at *P*≤.05.

### Ethical Considerations

The study was approved by the Psychology Research Ethics Committee of Leiden University (CEP19-1125/557). Furthermore, the study was preregistered through the Center for Open Science [32]. Before the start of the study, participants were asked to sign an informed consent form. After completing all the questionnaires, they were debriefed and provided with a few examples of real internet-based stress management interventions in case they needed one. As compensation, participants received course credits.

## Results

### Demographic Characteristics

A total of 146 students participated in our study and completed the questionnaire. Their mean age was 21.8 (SD 4.51) years, 103 (70.5%) were female, and 104 (71.2%) were of Dutch nationality (Table 1). There were no significant differences in demographic characteristics between the 2 groups.

**Table 1:** Baseline demographic characteristics.

Variable		Total sample (n=146)	Human-supported condition (n=73)	Self-help condition (n=73)
Age (years), mean (SD)		21.8 (4.5)	22.0 (4.6)	21.6 (4.4)
Female, n (%)		103 (70.5)	47 (66.2)	56 (76.7)
**Nationality, n (%)**				
	Dutch	104 (71.2)	49 (67.1)	55 (75.3)
	European (non-Dutch)	37 (25.3)	20 (27.4)	17 (23.3)
	Other	5 (3.4)	4 (5.5)	1 (1.4)

### Use Intention Per Condition

We found no significant difference in use intention between the human-supported condition and self-help condition (t_142_=–1.133; *P*=.26; Table 2). Furthermore, we found no differences between the 2 conditions in any of the other constructs (Table 2).

**Table 2:** Mean scores and SDs of use intention and its predictors.

Variable (scoring range)	Human-supported condition (n=73), mean (SD)	Self-help condition (n=73), mean (SD)	*P* value
Use intention (3-15)	7.5 (3.6)	8.2 (3.6)	.26
Expected working alliance (12-60)	42.3 (8.2)	40.3 (8.7)	.16
Performance expectancy (4-20)	13.8 (2.9)	14.0 (2.5)	.69
Effort expectancy (4-20)	16.9 (2.4)	16.8 (2.3)	.94
Social influence (4-20)	12.5 (2.9)	12.9 (3.1)	.66

### Working Alliance and Use Intention

**Figure 2:**
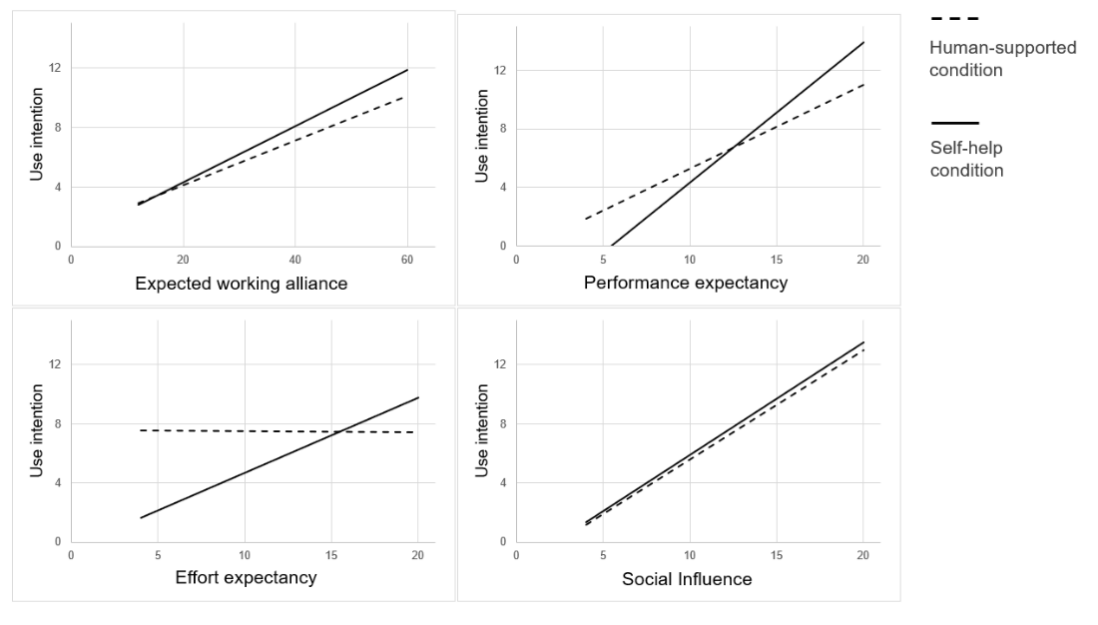
Simple slopes of the effects of expected working alliance, performance expectancy, effort expectancy, and social influence on use intention.

The GLM showed no significant association between condition and expected working alliance (*F*_1,140_=0.051; *P*=.82; η^2^=0). However, we did find a significant positive association between expected working alliance and use intention (*F*_1,140_=26.435; *P*<.001; η^2^=0.159). We found no significant interaction effect of condition and expected working alliance on use intention (*F*_1,140_=0.367; *P*=.55; η^2^=0.003; Figure 2).

### UTAUT Constructs and Use Intention

The GLM showed no significant association between condition and performance expectancy (*F*_1,140_=3.34; *P*=.07; η^2^=0.024). We did, however, find a significant positive association between performance expectancy and use intention (*F*_1,140_=69.269; *P*<.001; η^2^=0.331) and a significant interaction effect of condition and performance expectancy on use intention (*F*_1,140_=4.363; *P*=.04; η^2^=0.030). An increase in performance expectancy was related to a greater increase in use intention in the self-help condition compared to the human-supported condition (Figure 2).

We also found no significant association between condition and effort expectancy (*F*_1,140_=3.4086; *P*=.07; η^2^=0.024). However, again, we did find a significant positive association between effort expectancy and use intention (*F*_1,140_=3.961; *P*=.049; η^2^=0.028) and a significant interaction effect of condition and effort expectancy on use intention (*F*_1,140_=4.102; *P*=.045; η^2^=0.028). An increase in effort expectancy was related to a greater increase in use intention in the self-help condition but not in the human-supported condition (Figure 2).

Again, we found no significant association between condition and social influence (*F*_1,140_=0.003; *P*=.96; η^2^=0). We did find a significant positive association between social influence and use intention (*F*_1,140_=90.025; *P*<.001; η^2^=0.391) but this time we found no significant interaction effect of condition and social influence on use intention (*F*_1,140_=0.020; *P*=.89; η^2^=0; Figure 2).

## Discussion

### Overview

**Figure 3:**
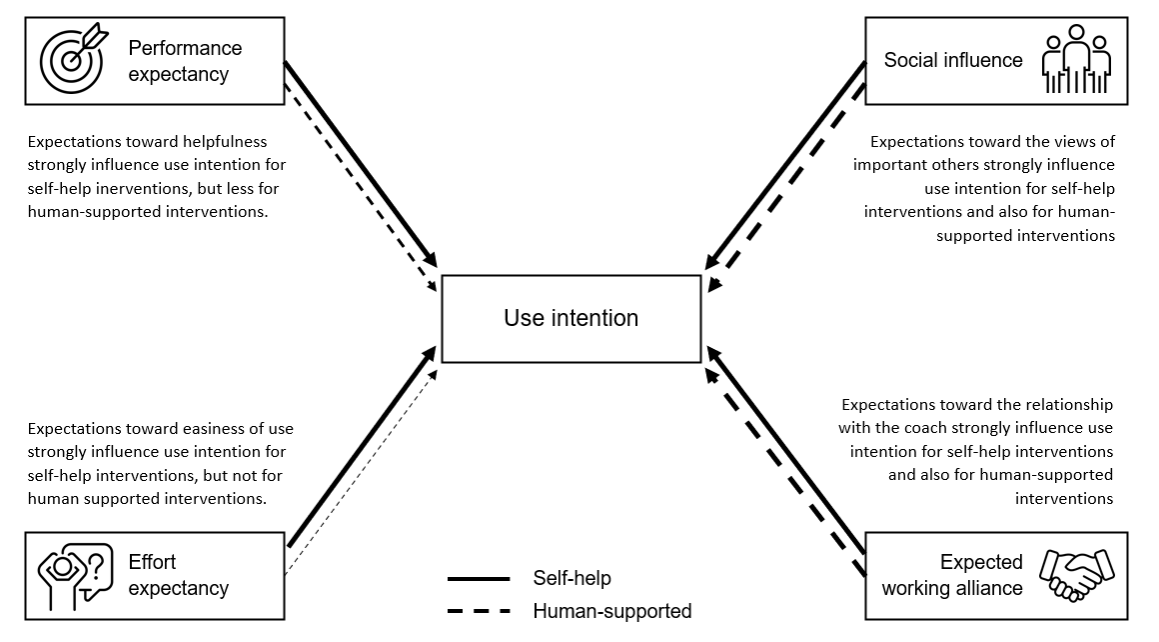
Overview of study findings.

In our study, we asked university students to evaluate a sham stress management app. We aimed to investigate whether there is a difference in use intention for self-help eHealth interventions compared to human-supported ones and what user expectations may influence this. We found that people were as likely to start using a self-help eHealth intervention as an eHealth intervention with human support. More than with human-supported interventions, the perception that the intervention might be ineffective or difficult to use limits the intention to start using self-help interventions. See Figure 3 for an overview of the findings.

Although previous studies show a relatively low uptake and use intention of self-help eHealth interventions [11-13], we did not find differences in use intentions between the self-help and human-supported interventions. Possibly, the health beliefs, perceptions, and skills of our student sample might have played a role in this [33]. Not only do perceptions about the effectiveness or ease of use of an eHealth tool affect the start of an intervention but also perceptions about the risks of getting health-related problems and actually performing the health-promoting behavior [34]. Furthermore, a younger age and higher educational level are related to a higher intention to start eHealth interventions in general [13]. Our sample might therefore have been more open to using eHealth interventions and were less influenced by the presence, or lack thereof, of human support. Future research could focus on investigating the role of age and educational level on use intentions of self-help and human-supported eHealth interventions. Another explanation for the differences in findings between our and previous studies [11,12] could be the use of different outcome measures. Although the UTAUT model predicts that use intention can predict actual use, studies do show that people have difficulties translating their intentions into actual behavior [35]. The objective measure of uptake might therefore have led to different results compared to the more subjective measure of use intention we used, which would be interesting to additionally take into account. Finally, the study that did find a difference in use intention between self-help and human-supported interventions focused on interventions for mental health, such as depression [13]. It would be interesting to test if the need for social support during eHealth interventions depends on the goal of the intervention (eg, psychological vs lifestyle improvements).

Interestingly, we found that an expected working alliance has an equally strong effect on the intention to use either a human-supported or self-help intervention. This result is in line with previous studies showing a positive effect of working alliance on intervention effectiveness and adherence, both within human-supported [21,22] and self-help eHealth interventions with automated support [23-25]. Our findings show that working alliance is not important only during an intervention but even before the intervention has started in the form of expectations. The similar effect of the expected working alliance in both conditions suggests that people not only are able to actually have relationships with technology [26] but also seem to expect building one with the technology they are about to interact with. These results would also mean that improving the expected working alliance before the start of an intervention (eg, by designing a digital character that would welcome the user) would be a way to possibly increase the uptake of self-help eHealth interventions.

Finally, we found that performance and effort expectancy had a stronger effect on the use intention of self-help interventions compared to human-supported interventions. Not only the UTAUT model but also models such as the Health Belief model show that perceived benefits and perceived barriers affect whether people start with a health-promoting behavior, such as stress management [33]. What is new, though, is that the perceived effectiveness and ease of use of the intervention have a more pronounced impact on intention to use an intervention for interventions with an absence of human support compared to interventions where human support is available. This suggests that the perception that the intervention might be ineffective or difficult to use diminishes the intention to start using a self-help intervention but not the intention to start using a human-supported intervention. Meta-analyses show that the mere presence of a human being (even a nonprofessional) is a key ingredient in intervention effectiveness and the prevention of dropout [36-38]. Just the option of having someone available to provide procedural support (related to performance expectancy) or technical support (related to effort expectancy) seems to be enough for people to be motivated to start something new. The presence of a human coach could act as a buffer against negative expectations, which would make it easier for these people to adhere to the intervention [39]. Possibly, the mere presence of social support in the human-supported intervention could compensate for a lack of self-efficacy (the extent to which one believes in his or her own capabilities [40]) that people may feel when using a new intervention [41,42]. This could lower the perceived barriers and increase willingness to start using the intervention [33]. Exploring this further is crucial in a clinical context because individuals with limited social support tend to experience reduced adherence to health interventions and demonstrate less favorable intervention outcomes [39,43]. Even despite the relatively high use intention of self-help eHealth interventions, these results indicate that it is important to take the user’s needs and wishes into account when deciding on the level of human support to provide during an intervention.

Self-help eHealth interventions will become more and more important in health care practice. To ensure uptake of new eHealth interventions, professionals could screen the user’s expectations toward the intervention’s helpfulness and ease of use beforehand (Table 3). If the user’s expectations turn out to be low, it would be useful to incorporate some level of human support into the eHealth intervention to prevent people from dropping out even before the start of the intervention. Additionally, designers of self-help eHealth interventions could pay extra attention toward its perceived helpfulness and ease of use. Preventing negative user expectations toward the intervention’s performance or effort expectancy could help increase the uptake of self-help eHealth interventions.

**Table 3:** Items of the Unified Theory of Acceptance and Use of Technology subscales: performance expectancy (PE) and effort expectancy (EE).

Item	Statement
PE1	I find [name eHealth technology] useful.
PE2	Using [name eHealth technology] enables me to [target behavior].
PE3	Using [name eHealth technology] will [target behavior].
PE4	If I use [name eHealth technology] I will know how to [target behavior].
EE1	My interaction with [name eHealth technology] is clear and understandable.
EE2	It would be easy for me to develop the skills needed to use [name eHealth technology].
EE3	I think [name eHealth technology] would be easy to use.
EE4	It would be easy to learn how to operate [name eHealth technology].

### Strengths and Limitations

Our study was not without limitations. For example, although the screenshots of the app were adjusted to the experiences and interests of our sample, it is plausible that the topic of stress management was not equally relevant for all students, which could also have affected use intentions. For future studies, it would be better to tailor the goal of the eHealth intervention (eg, decreasing stress or improving physical activity) to the actual interests of the individual participants to investigate if and how this affects a participant’s use intention. Second, we used a university student population to test our hypotheses. People with a younger age and higher educational level have a more favorable attitude toward eHealth interventions in general [13]. To be able to generalize our findings, future research should investigate whether the same effects are found in other populations. It would be interesting to replicate this study with a target population who would benefit the most from eHealth interventions, for example, older patients with a chronic disease, to see if their expectations toward either human or automated support have similar effects on their intention to start with such interventions.

### Conclusions

In our study, we investigated what expectations drive the intention to start using self-help and human-supported eHealth interventions. The results suggest that expectations toward the intervention’s helpfulness and ease of use are especially relevant regarding the use of self-help interventions. This means that people who have doubts about the intervention’s usefulness or usability would benefit the most from additional human support. The question, however, remains whether such expectations are also relevant for actual uptake. Our study provides a basis to further investigate user expectations within a clinical sample, which will provide health care practitioners with the tools to influence the uptake of eHealth interventions.

## Appendix

Multimedia Appendix 1 Screenshots shown in human-supported and self-help conditions.

Multimedia Appendix 2 CONSORT-eHEALTH checklist (V 1.6.1).

## Abbreviations

GLM: general linear model
UTAUT: Unified Theory of Acceptance and Use of Technology
WAI-SR: Working Alliance Inventory–Short Revised form
