# Supplementary material for: Use Intention and User Expectations of Human-Supported and Self-Help eHealth Interventions: Internet-Based Randomized Controlled Trial
Source: JMIR Form Res. 2024 Feb 15;8:e38803. doi: 10.2196/38803 (PMC10905349; doi:10.2196/38803)
Supplement: Multimedia Appendix 1 [file formative_v8i1e38803_app1.docx]

APPENDIX 1.

Screenshots shown in human-supported and self-help condition.

|  | Condition | |
| --- | --- | --- |
|  | Human-supported | Self-help |
| Loading screen | 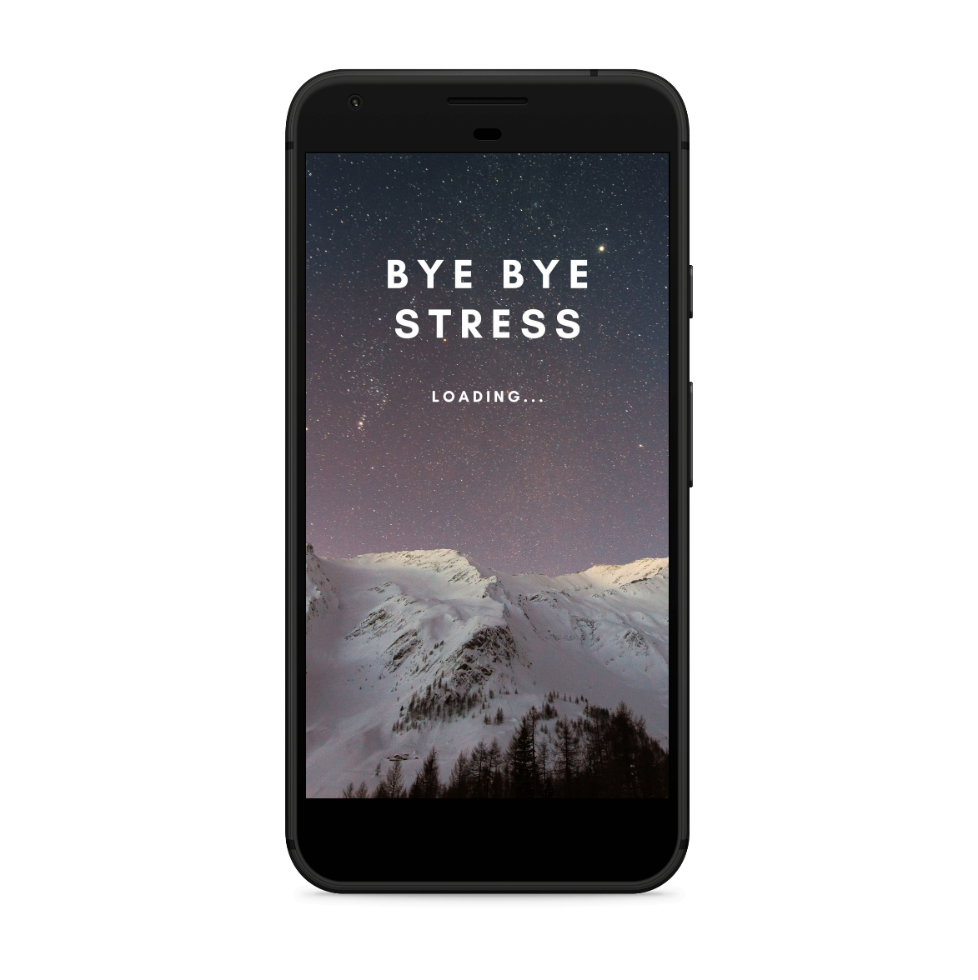 | |
| Welcome screen | 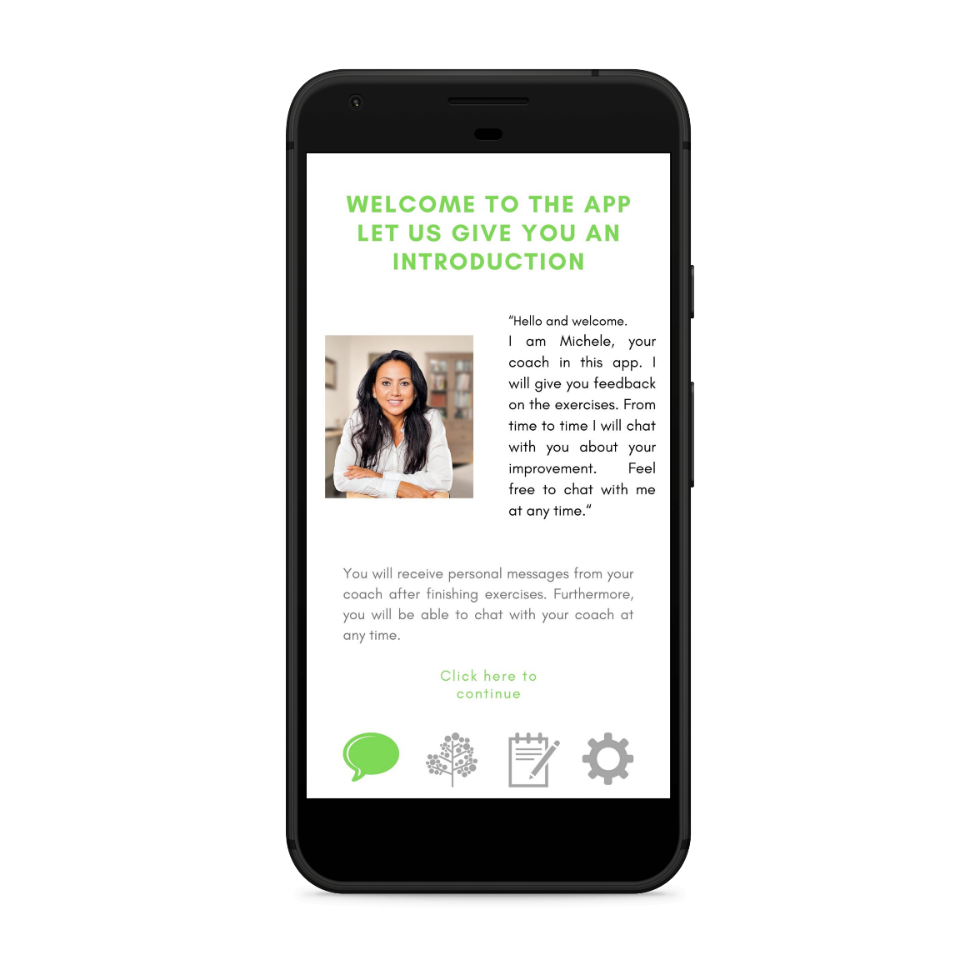 | 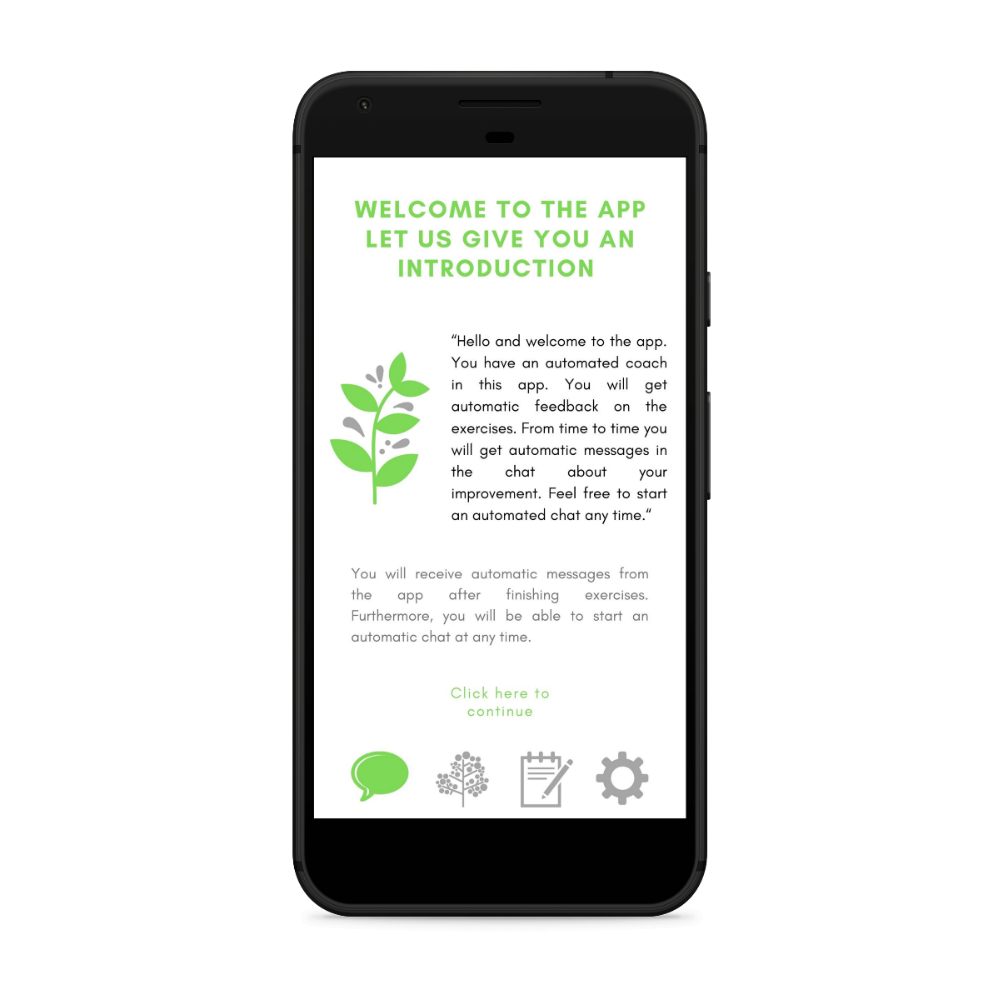 |
| App menu | 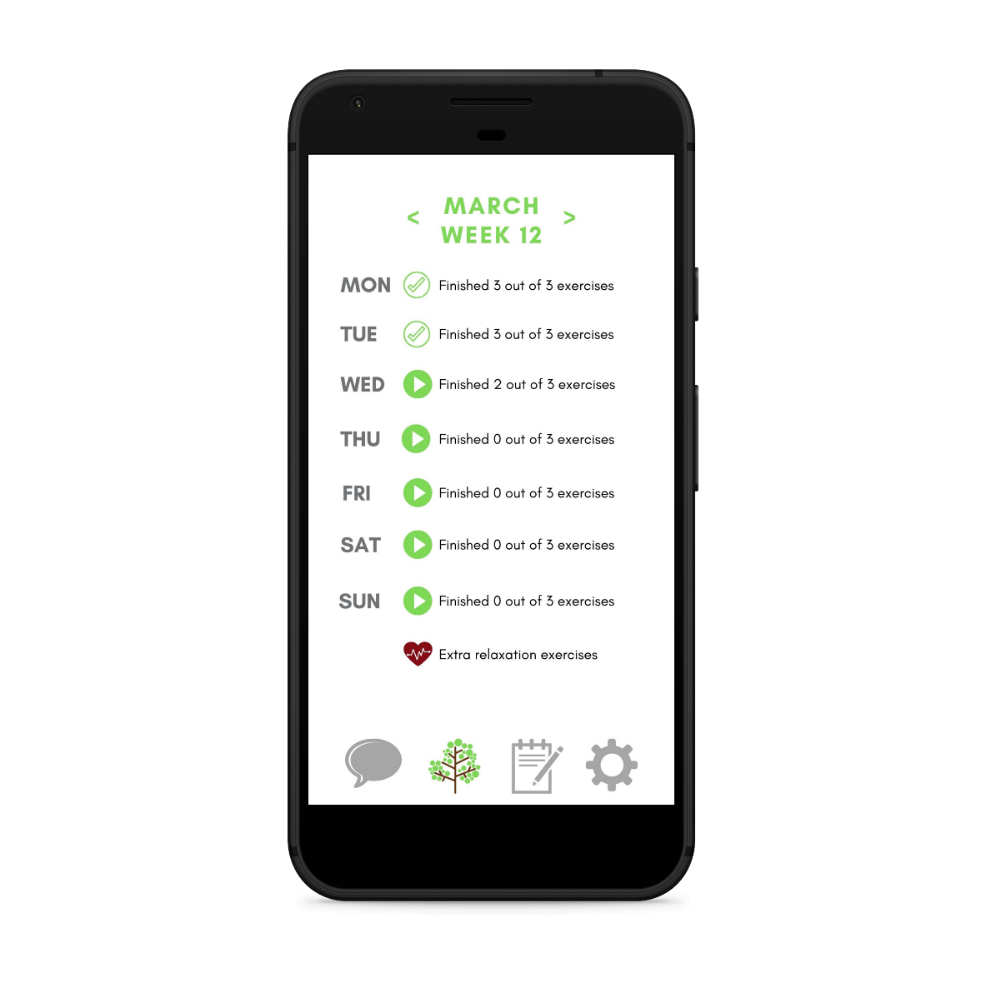 | |
| Overview messages | 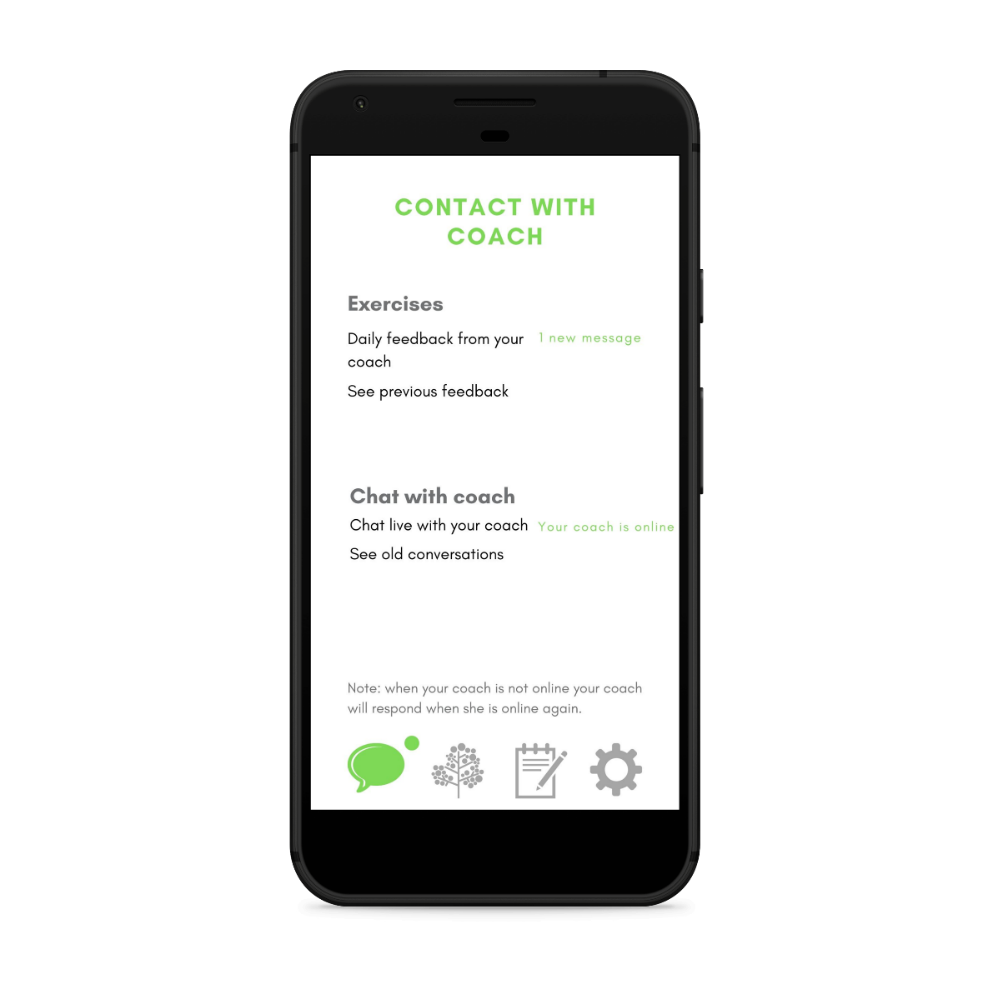 | 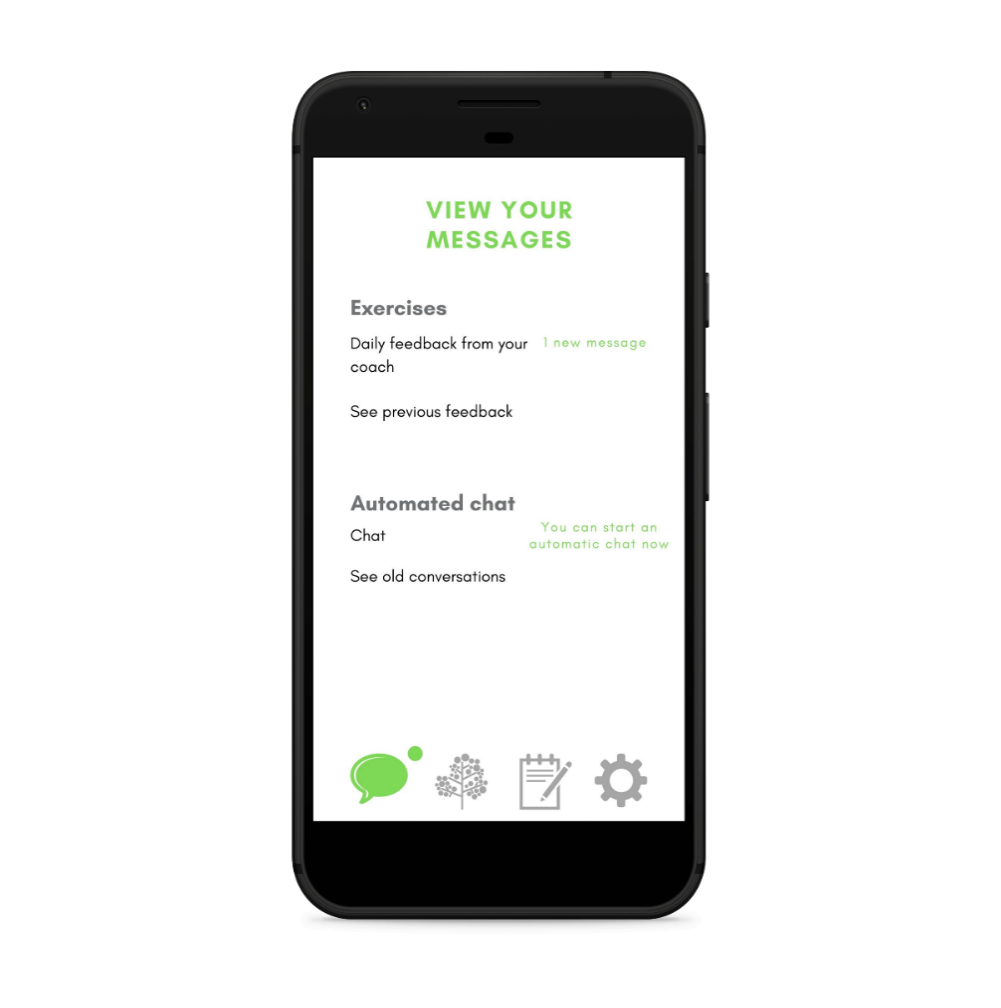 |
| New message | 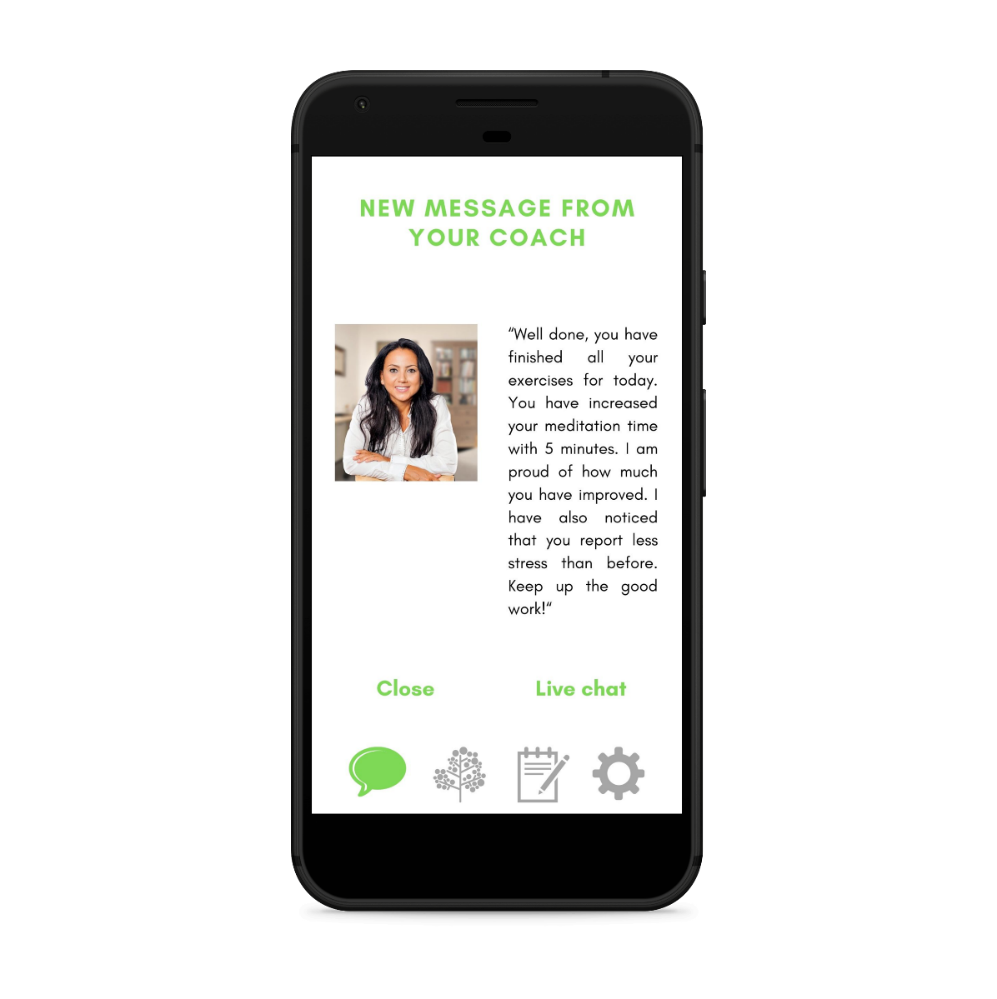 | 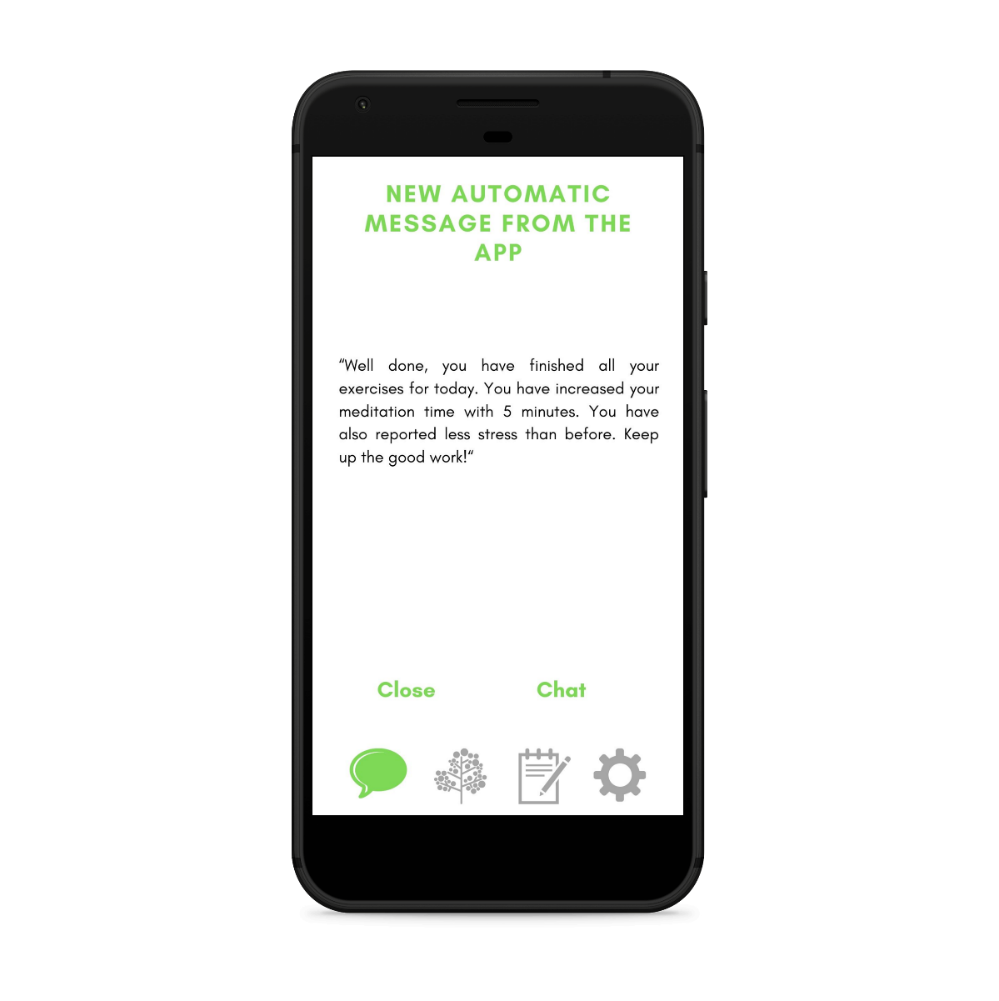 |
| Chat | 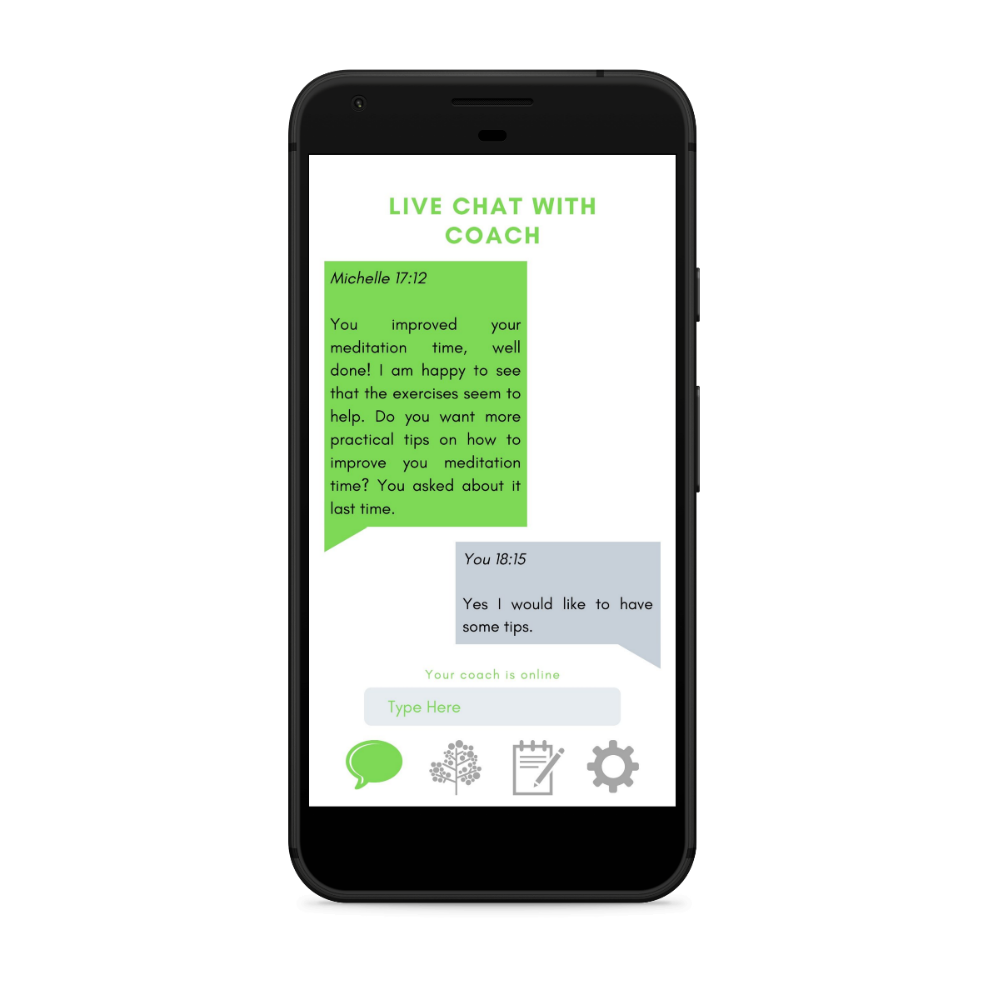 | 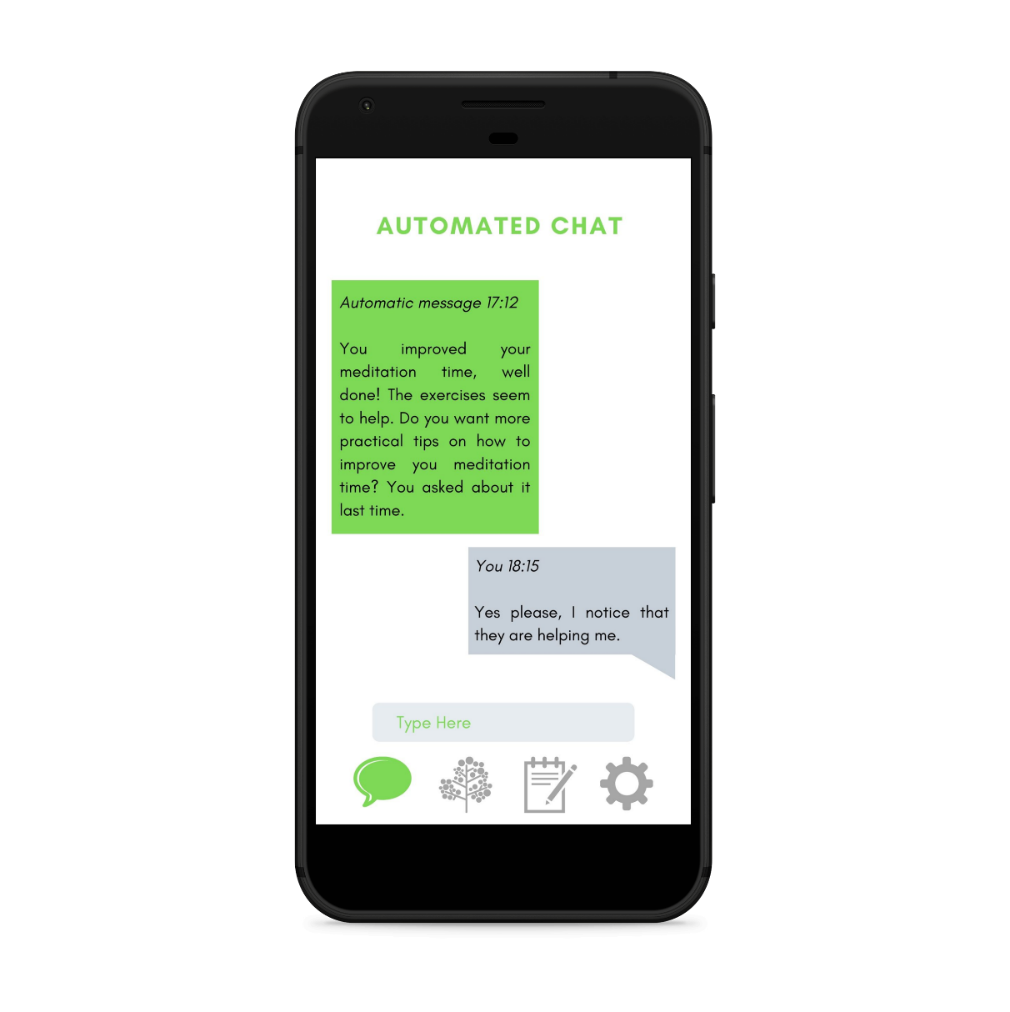 |
| Exercise completion | 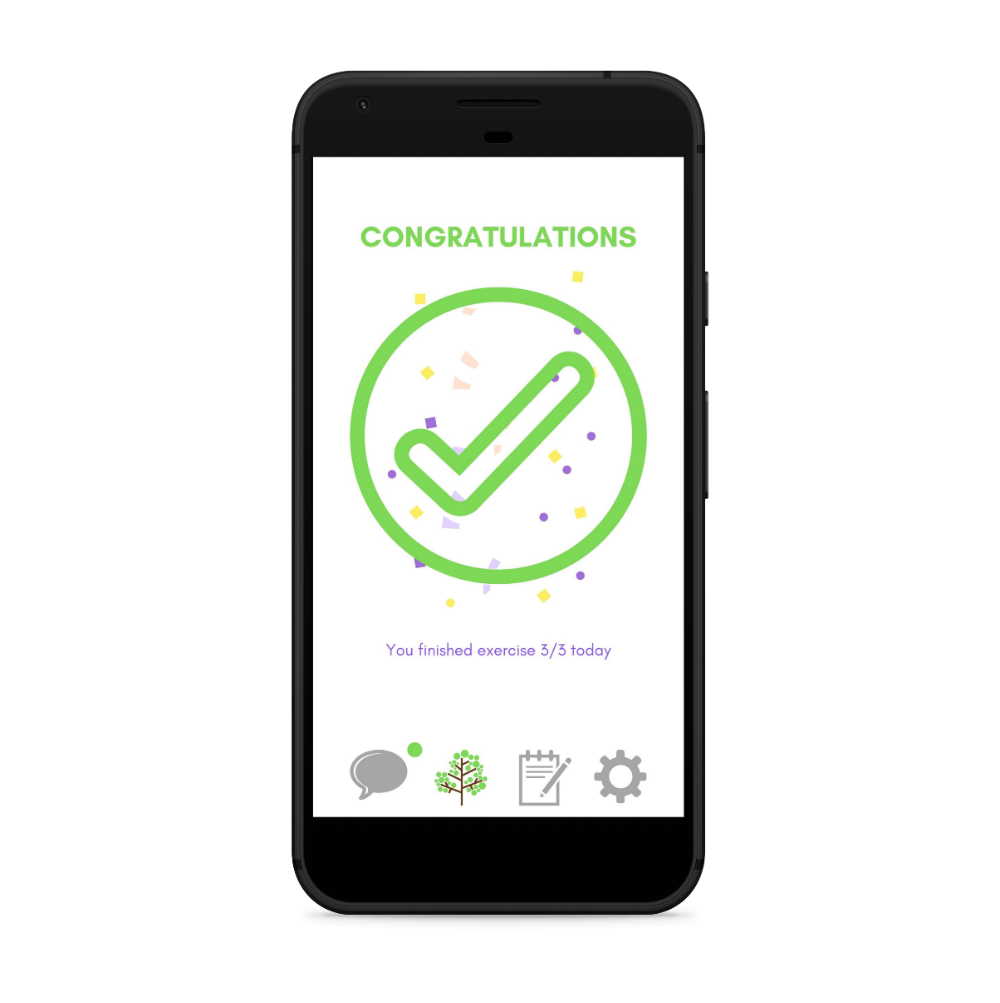 | |
| Progress overview | 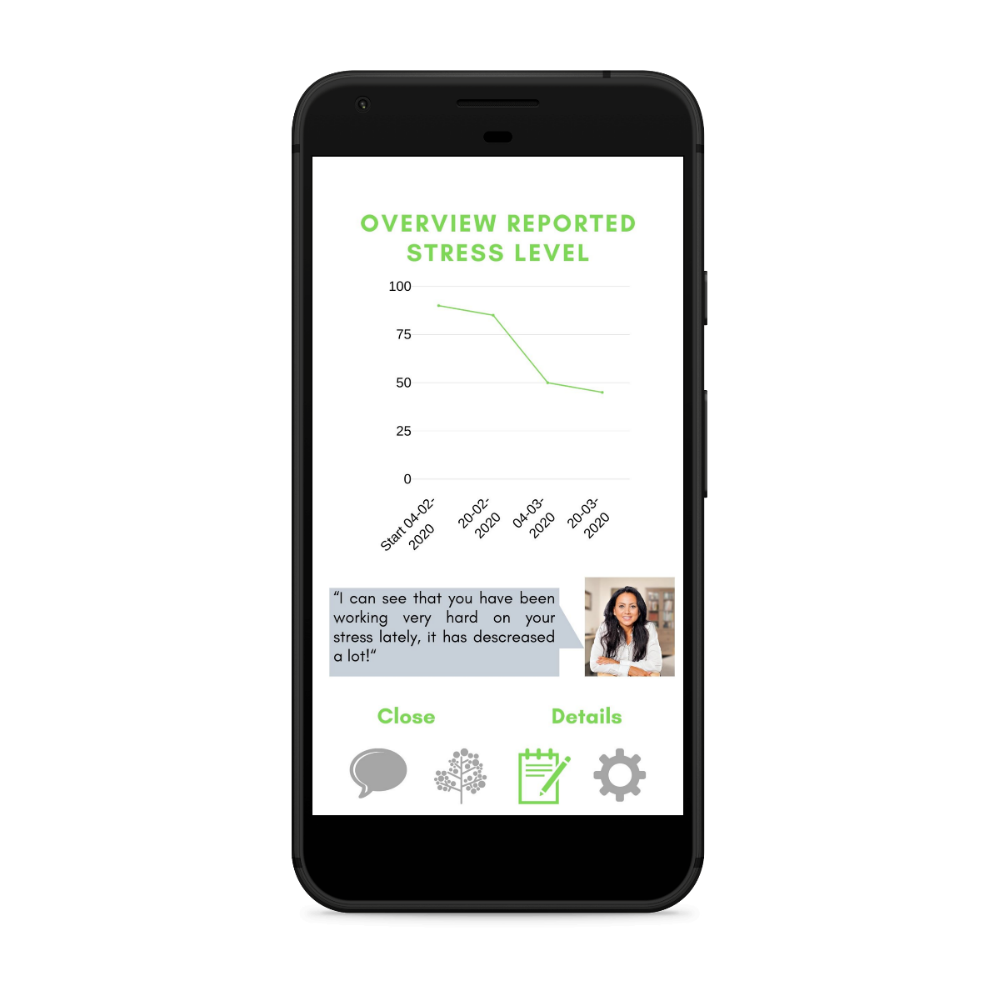 | 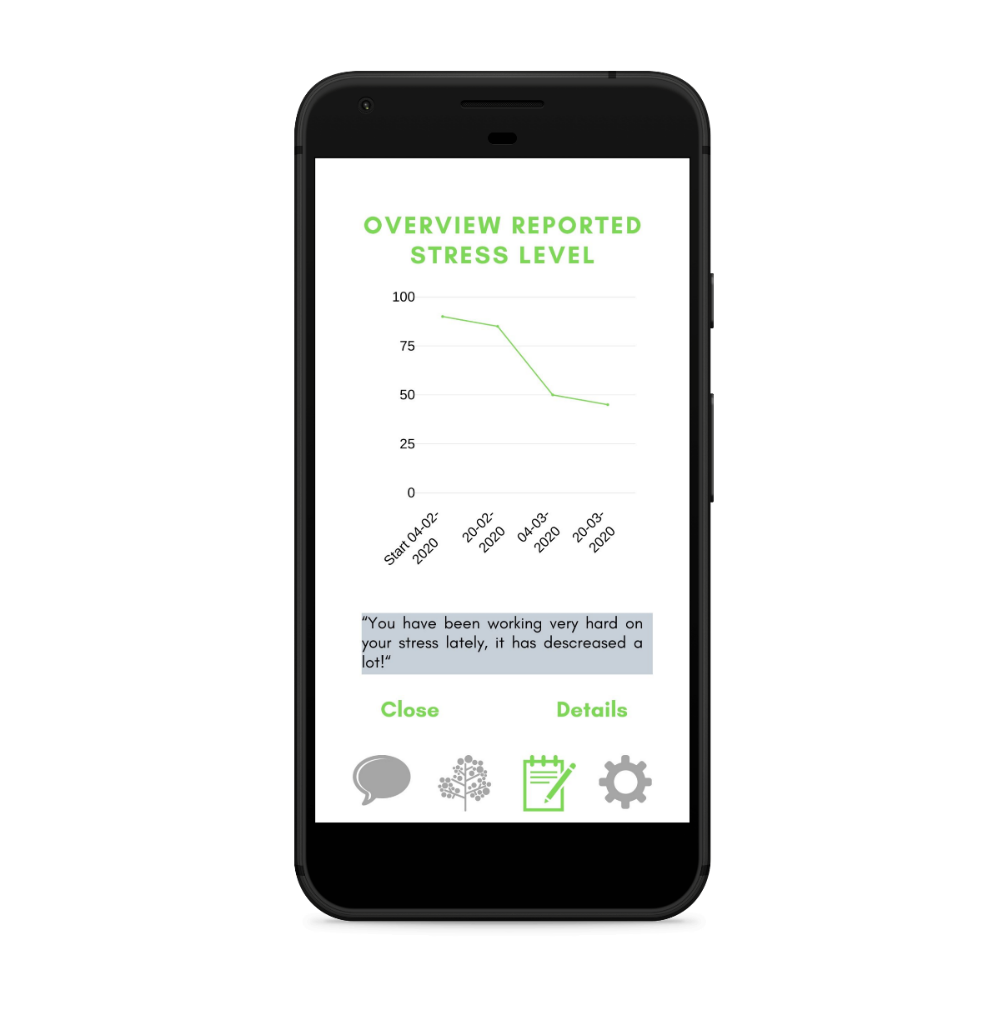 |
| Settings | 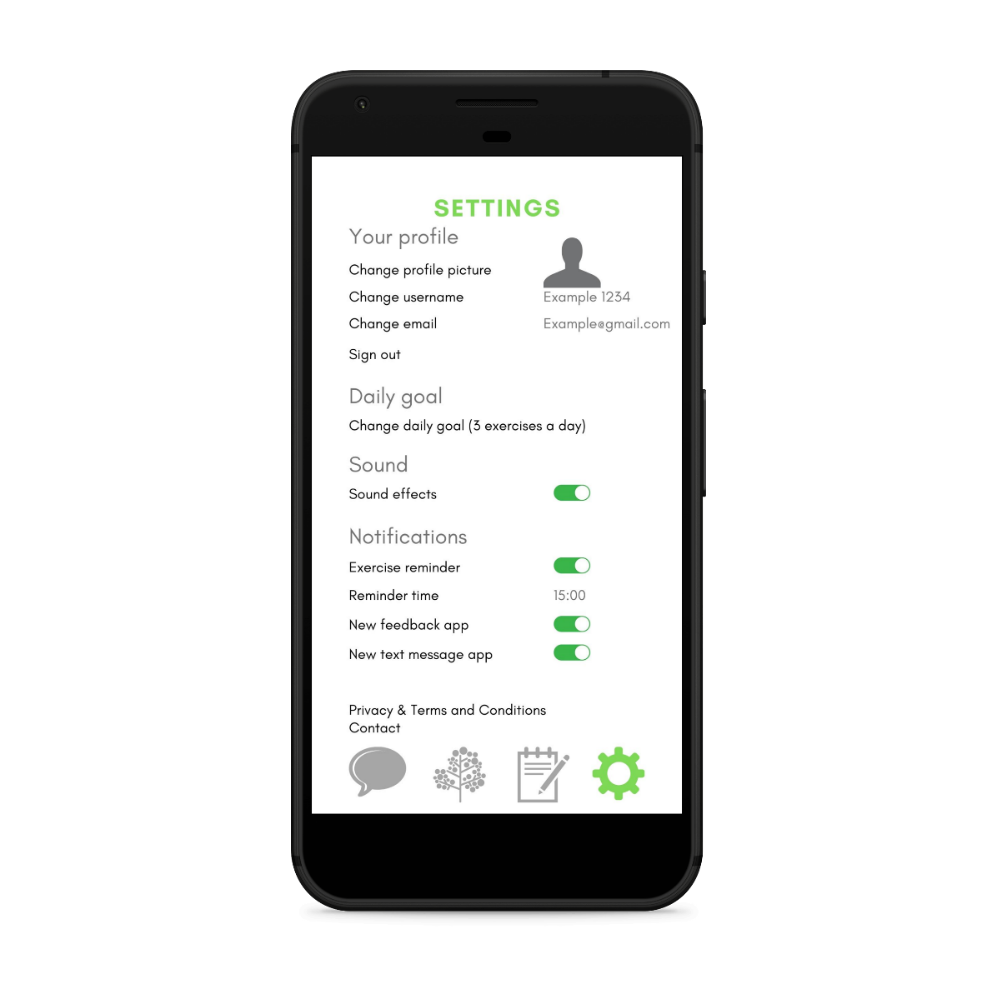 |  |
